# Reimagining clinical psychology for a changing planet

**DOI:** 10.1111/papt.70050

**Published:** 2026-05-14

**Authors:** Georgia King, Matthew Adams, Nick Maguire, Marc Williams

**Affiliations:** ^1^ School of Clinical Psychology University of Southampton Southampton UK; ^2^ School of Humanities and Social Science University of Brighton Brighton UK; ^3^ School of Psychology Cardiff University Cardiff UK

**Keywords:** climate and ecological emergencies, climate change, clinical psychlogy, mental health, story stem

## Abstract

**Objectives:**

The interconnected climate and ecological emergencies (CEE) are driving irreversible planetary changes with profound consequences for physical and mental health. Clinical psychology holds valuable skills for mitigating and adapting to these impacts, yet the profession lacks a coherent vision for its role. This study aimed to generate such a vision.

**Design:**

A qualitative design using individual and group story completion tasks explored imagined roles for clinical psychology in responding to the CEE.

**Methods:**

Forty‐nine practitioner psychologists and/or climate activists completed online story stems individually, and 28 participated in group‐based story co‐creation workshops. Thematic analysis was used to identify shared themes across individual and group narratives.

**Results:**

Three overarching themes captured the imagined role of clinical psychology: (1) *Acknowledgement*—recognising the root causes of the CEE and associated emotional responses; (2) *Engagement*—supporting clients to engage with distress, rethinking mental health through nature‐based and socially engaged perspectives and (3) *Transformation*—collaborating with communities, supporting climate activism, embracing political roles and fostering imagination.

**Conclusions:**

The findings offer a theory‐informed vision of psychological adaptation to ecological crisis. Clinical psychology can expand its remit to include climate‐responsive roles spanning grief work, eco‐therapy, activism and systems‐level change. This novel application of story completion methodology surfaces imaginative but plausible directions for the profession in facing the CEE.

## INTRODUCTION

We live in a period marked by irreversible changes to Earth's life systems caused by human activity, threatening both planetary and human health (Whitmee et al., 2015); this is often termed the *climate and ecological emergencies* (CEE). The UK Government now recognises that ‘the climate crisis is a health crisis’ (UK Health Security Agency, 2023). The planetary health framework has been developed by an international taskforce of leading thinkers to underscore the interdependence of human and environmental wellbeing (Myers & Frumkin, 2020), identifying nine boundaries essential for sustaining human activity—six of which have already been breached (Rockström et al., 2009; Steffen et al., 2015).

These breaches have profound consequences for mental, physical and community health. Rising temperatures are associated with increased rates of suicide (Thompson et al., 2018), hate speech (Stechemesser et al., 2022) and use of mental health services (Vida et al., 2012). Climate‐related events such as flooding, storms and wildfires are linked to elevated levels of depression, anxiety and post‐traumatic stress, often due to displacement and community disruption (Cruz et al., 2020; Finlay et al., 2012; Graham et al., 2019). Sea level rise is expected to cause further displacement (McLeman, 2018), while broader climatic shifts threaten global food security (Wheeler & Von Braun, 2013; Ziska et al., 2016) and may fuel conflict (Martin‐Shields & Stojetz, 2019).

These impacts disproportionately affect those least responsible for climate change—a ‘double injustice’ (Gough, 2011; Ingle & Mikulewicz, 2020; Watts et al., 2018). Children and young people, in particular, are vulnerable. Many experience distress in response to the CEE (Lawrance et al., 2022), with some researchers suggesting this is affecting their sense of safety and relational attachments (Sanson & Burke, 2020). Young people in the UK and internationally report outrage at perceived governmental inaction (Hickman et al., 2021) and experience a range of emotional responses to both the direct and indirect consequences of the CEE (Lawrance et al., 2021). Parents and caregivers, too, may experience moral injury as they attempt to protect children on a planet under threat (Bechard et al., 2023).

Given the scope of these impacts, psychologists are increasingly seen as key actors in conceptualising and responding to the CEE (Clayton et al., 2016; Li et al., 2022; Swim et al., 2011). In the UK, practitioner psychologists are expected to promote health, prevent ill‐health and understand the broader determinants of wellbeing (Health & Care Professions Council, 2023). Internationally, the World Health Organization has called for expanded mental health provision and psychosocial support in light of the CEE (World Health Organization, 2022). Despite this, some clinical psychologists report feeling professionally constrained or unprepared to respond (Stilita & Charlson, 2024; Walker & Lloyd, 2025). This is echoed by recent findings that mental health practitioners perceive a lack of structured support or ‘helpline’—highlighting the absence of service‐level pathways for climate distress (Williams et al., 2024), while the broader public remain silent amid a ‘climate spiral of silence’ (Maibach et al., 2016; Noelle‐Neumann, 1993).

There are signs that this silence is shifting. In the UK, professional psychological bodies have begun explicitly encouraging engagement with the CEE (British Psychological Society, 2023; Morgan et al., 2020; Williams & Bennetts, 2024), while global initiatives like PsyFuture (n.d.) and the Climate Psychology Alliance (n.d.) are fostering international collaboration. Mental health practitioners (MHPs) report growing numbers of clients presenting with climate distress (Croasdale et al., 2023). It has been suggested that MHPs must extend their roles beyond individual therapy to include climate‐attuned assessment, workforce support and community advocacy (Crandon et al., 2022). Yet in practice, many psychologists remain disengaged from climate mitigation or feel ill‐equipped to support climate‐related mental health issues (Stilita & Charlson, 2024), perhaps due to a lack of vision for how clinical psychology might contribute meaningfully in this space.

In parallel, researchers in energy and climate science are increasingly turning to imaginative methods to disrupt business‐as‐usual thinking and cultivate new possibilities (Brown, 2017; Moezzi et al., 2017). Counterfactual worlds, where participants imagine alternate present‐day realities, can liberate thinking from current constraints (Hillgren et al., 2020). Similarly, the concept of ‘radical imagination’ encourages envisioning positive alternative futures that can guide action (Scurr & Bowden, 2021). In climate psychology, such approaches can move people away from despair and towards active hope—the belief that positive change is possible through agency (Macy & Johnstone, 2022; Marks et al., 2023; Ward, 2023). For example, Finnerty et al. (2025) used a critical discursive psychology approach to analyse how environmentally concerned scientists imagined the future, showing that more open, transformable constructions of the future enabled a broader repertoire of hopeful and solution‐focused responses.

Story completion tasks have emerged as a promising imaginative method, particularly within qualitative research led by Braun and Clarke (Braun et al., 2019; Clarke & Braun, 2019; Clarke & Moller, 2017). In these tasks, participants are given the beginning of a story (the stem) and asked to complete it. This projective technique allows insight into participants' values, beliefs and assumptions—especially around contentious issues like the CEE (Braun & Clarke, 2013; Clarke et al., 2019). Braun et al. (2019) have called for researchers to explore collectively written stories, aligning with traditions of storytelling and the ethos of collective climate action. Collective story generation resonates with interdisciplinary collaboration and deliberative practices like climate assemblies (Devaney et al., 2020; Milman et al., 2017). It is through imaginative collaboration that the complex survival of humanity might be re‐envisioned (Harris et al., 2010).

This study aimed to explore how clinical psychology could respond to the CEE through both mitigation and adaptation. By using individual and group‐based story completion tasks, the research elicited imagined futures from practitioner psychologists and climate activists. In doing so, it offers insight into how the profession might evolve to meet the mental health challenges posed by ecological crises. The findings contribute to emerging theory on the role of clinical psychology in collective, systemic threats, while also informing therapeutic thinking, professional identity and service design in a time of planetary disruption.

## METHODS

### Ethics

Ethics approval was granted by the University of Southampton Research Ethics Committee (79196).

### Phase 1: Story stem completion task

#### Sampling and recruitment

To be eligible to take part in the study, participants had to be over 18 years of age, have access to the internet, self‐identify as either a practitioner psychologist or a climate activist and endorse the statement that ‘the climate and ecological emergency impacts mental health’. Practitioner psychologists include the protected titles of: Practitioner psychologist, Registered psychologist, Clinical psychologist, Forensic psychologist, Counselling psychologist, Health psychologist, Educational psychologist, Occupational psychologist and Sport and Exercise psychologist, as regulated by the Health Care Professional Council (Gledhill, 2023). Those undertaking training to become a practitioner psychologist, trainee practitioner psychologists, were also included. The study was advertised online via special interest groups for climate‐concerned psychologists and activists and at associated events, as well as via the school of psychology at the University of Southampton. Recruitment took place between the 30th of June 2023 and the 27th of December 2023.

Both practitioner psychologists and climate activists were recruited because each brings unique but complementary insights: psychologists offer expertise in human behaviour and wellbeing, while climate activists bring lived experience and a deep understanding of environmental challenges. Analysing their stories together, rather than separately, allowed these perspectives to intersect and inform one another, generating a richer, more collaborative vision than either group could have produced alone.

#### Procedure

Study adverts contained a link to an online survey platform where participants were presented with the participant information sheet and consent form. After providing written informed consent to take part, participants were presented with two videos. The first explained the mental health impacts that the CEE are having on people. The second explained the role of a clinical psychologist. This was to ensure a basic level of knowledge was shared between the participants before taking part.

Participants then completed the story completion task, in which they were presented with the following story stem:

It's 2070 and Ashley has reached old age. Ashley is reminiscing about how they were part of shaping and informing green practices in clinical psychology. This contributed to bringing human and non‐human life back from the brink of collapse.

Participants were asked to:

Describe Ashley's journey and any developments within clinical psychology, based on the idea that human mental health is connected to the health of the planet.

There was a minimum character count (including spaces) of 2500 for story stem responses, to encourage parity between written stories; this was based on prior piloting and feedback.

After providing an answer to the story completion task, participants provided demographic information (age, gender, country of residence and ethnicity) and completed two measures: the New Ecological Paradigm Scale (Dunlap et al., 2000) and the Values Orientation Scale (De Groot & Steg, 2008), to gain a picture of the sample's environmental worldview and values, respectively. Finally, participants were presented with a debrief page.

#### Participants

A total of 51 individual stories were recorded, two of which were deemed ineligible and thus removed. This was because one participant copied and pasted a short paragraph to make up the minimum character length requirement and the other participant wrote their story from a climate denial perspective, that the climate movement is alarmist, which contravened consent parameters; thus, both were removed. This resulted in 49 participants (36 females; 11 males; 2 other) for phase one. This is in line with sample size recommendations for story completion methodology (Clarke & Braun, 2013). There were 22 practitioner psychologists and 27 climate activists, with a participant mean age of 41.33 years old (SD = 19.89, range = 18–77). All practitioner and trainee psychologists were 25 years old or above and would have undergone extensive undergraduate and postgraduate training courses to be awarded such titles. As such, the psychological competencies within this population were considered homogeneous. All participants were allocated pseudonym initials (see Table S1 for participant demographics).

Two measures were used to gauge the participants' environmental beliefs and intentions. The Values Orientation Scale (De Groot & Steg, 2008) is a psychological measure used to assess people's environmental value orientations and demonstrates good overall psychometric quality. It identifies three core value orientations: biospheric values encompass concern for the environment and nature; altruistic values encompass concern for the well‐being of other people; and egoistic values encompass caring for one's own personal benefit. This scale resulted in participants scoring more highly for altruistic values (*M =* 6.06, SD = 0.95, range = 3.25–7.00) and biospheric values (*M* = 5.96, SD = 1.06, range = 2.75–7.00) in comparison to egoistic values (*M* = 1.44, SD = 1.01, range = −0.5 to 3.25). This suggests participants would be more likely to behave pro‐environmentally if it were to benefit others and the planet, as opposed to themselves.

The New Ecological Paradigm scale (Dunlap et al., 2000) is a well‐validated 15‐item scale used to measure whether participants' endorse ecocentric (i.e. see humans as part of nature) or anthropocentric (i.e. see humans as separate from nature) world views. It demonstrates good overall psychometric quality. Participants scored on average 4.21 (SD = 0.42, range = 3.13–4.93), which indicates that participants held high ecological world views, whereby they would consider humans to be part of the world's natural systems, as opposed to being superior to nature. Accordingly, this sample held strong environmental beliefs.

### Phase 2: Group workshop

A workshop was delivered as part of a British Psychological Society Climate Event on the topic of ‘Creating a collaborative vision for a future of planetary health’ on the 4th of December 2023. In this session, a group story completion task was held.

#### Sampling and recruitment

Members of the British Psychological Society who had already signed up to this event were offered the opportunity to take part in the second phase of this study.

#### Procedure

Attendees were emailed an information sheet and consent form before the workshop, which explained that the workshop would entail using story completion methodology as a means to answer difficult questions about the CEE for future generations. Attendees were informed that the written stories would be collected, and the verbal sharing of the stories would be audio‐recorded. It was made clear that consenting to take part in the research was not a prerequisite to attending the event. Attendees were free to consent or decline to take part in the research without it affecting their engagement in the event.

All attendees granted written informed consent on the day and were split into six groups of approximately five people per group. Each group was given the same story completion task as in phase one to complete. Participants were instructed to spend 15 min discussing their initial thoughts about how they would complete the story as a group, followed by 15 min capturing the co‐created story on paper. A member of each group then orally shared the co‐created story with the rest of the workshop, which was audio‐recorded. Written versions of the stories were collected and the audio recordings were later transcribed.

#### Participants

Twenty‐eight individuals over the age of 18 consented and took part in the workshop. Due to the nature of the live research event, demographics could not be collected for everyone. Of the 13 who provided demographics, the mean age was 50, 23% identified as male, 77% identified as female, 84% identified as White‐British, 8% as White‐Irish and 8% as White‐other.

### Data analysis

Qualitative material from participants was analysed using Reflexive Thematic Analysis (Braun & Clarke 2021) on both the individual and co‐created stories. A period of familiarisation was undertaken, whereby individual stories were read and recorded so that they could be listened to. Co‐created stories were also read and listened to. Once the data was familiar, the stories were coded line by line in relation to the research question, using NVivo software (2020). These stages were conducted blind, whereby participants' identity (i.e. practitioner psychologist or climate activist) was not known so to support the limitation of bias. This was important to ensure that no greater weight was given to the visions of practitioner psychologists or of climate activists. Conducting the analysis blind enabled the visions of the practitioner psychologists and the climate activists to converge, creating a collaborative vision mirroring participatory service development processes. However, it was known which stories were co‐created since the first author conducted the workshop and analysis.

To support the process of reflexivity within the analysis, PPI input was sought for the coding relevance as well as theme development. Two clinical psychologists, who also identify as climate activists, and one climate activist provided their perspectives on whether the developing themes felt meaningful and which themes resonated most with their personal experiences. Iterative versions of thematic maps supported the development of the final three themes. Throughout this process of analysis, supervision was held with the other researchers to support how the data was made sense of. These multiple processes supported the development of themes which were grounded in the data. Accordingly, quotes that best represented facets of the themes were chosen for illustrative purposes. Importantly, existing research and theory were also utilised to aid the interpretation process and develop the analytical narrative, since this pre‐existing understanding of such research and theory was the lens through which the first author understood the data. Therefore, elements of inductive and deductive approaches were taken to support the analysis and write‐up. As such, within the written results, the findings will be integrated with the wider literature it connects to. This is done to provide greater transparency for the interpretative work of the reflective thematic analysis undertaken (Braun & Clarke, 2021).

A critical realist approach was taken within the analysis, whereby ontological realism is combined with epistemological relativism (Bhaskar, 2013). This is because the first author (GK) considers the CEE to be a reality which cannot be avoided and one in which there are many different opinions as to how best to respond. It is understood that this positioning will likely have guided her engagement with the data. Thus, to support reflexivity within this process, a reflective journal was kept and utilised throughout the analysis process.

### Positionality statement

Accepting the realities of the CEE has led the first author (GK) to spend the last four years being active in climate activist groups as well as climate psychology groups. Advocating for planetary health within the profession of clinical psychology is part of her conceptualisation of what makes her a psychologist; it was from this position that the data were analysed. GK is a white‐British, middle‐class, cis woman, who, at the time of conducting the study, was a trainee clinical psychologist. She approached this topic from a position of relative privilege into an area where it is known that those who are least privileged are most affected by the CEE (Levy & Patz, 2015). GK believes that research can be used to highlight such inequities and it is in a similar vein that social and climate justice informs her activism. It is important to note that GK knew some of the workshop attendees from her professional networks.

## RESULTS

Throughout the analysis, it became clear that participants—whether psychologists or activists—did not imagine the role of clinical psychology in isolation, but embedded within broader systems of change. To reflect this interdependence, we drew on Bronfenbrenner's (1979) ecological theory, which conceptualises individuals as nested within overlapping systems of influence, from the interpersonal to the societal; and Voorhees and Perkins' (2007) planetary perspective which extends that conceptualisation to include natural environments and planetary health. Building on these theories, we structured our findings around four interlinked layers where participants imagined transformation across: (1) the role of the individual psychologist, (2) clinical psychology as a discipline, (3) wider society and (4) the planet. This layered framework allowed us to situate participants' visions within a holistic, system‐oriented understanding of change.

Three overarching themes were derived from the thematic analysis: (1) *Acknowledgement*, (2) *Engagement* and (3) *Transformation*. Each theme mapped across multiple ecological levels and described a stepped vision for how clinical psychology could help mitigate and adapt to the CEE. Figure 1 illustrates this multi‐layered model of reimagining.

**FIGURE 1:**
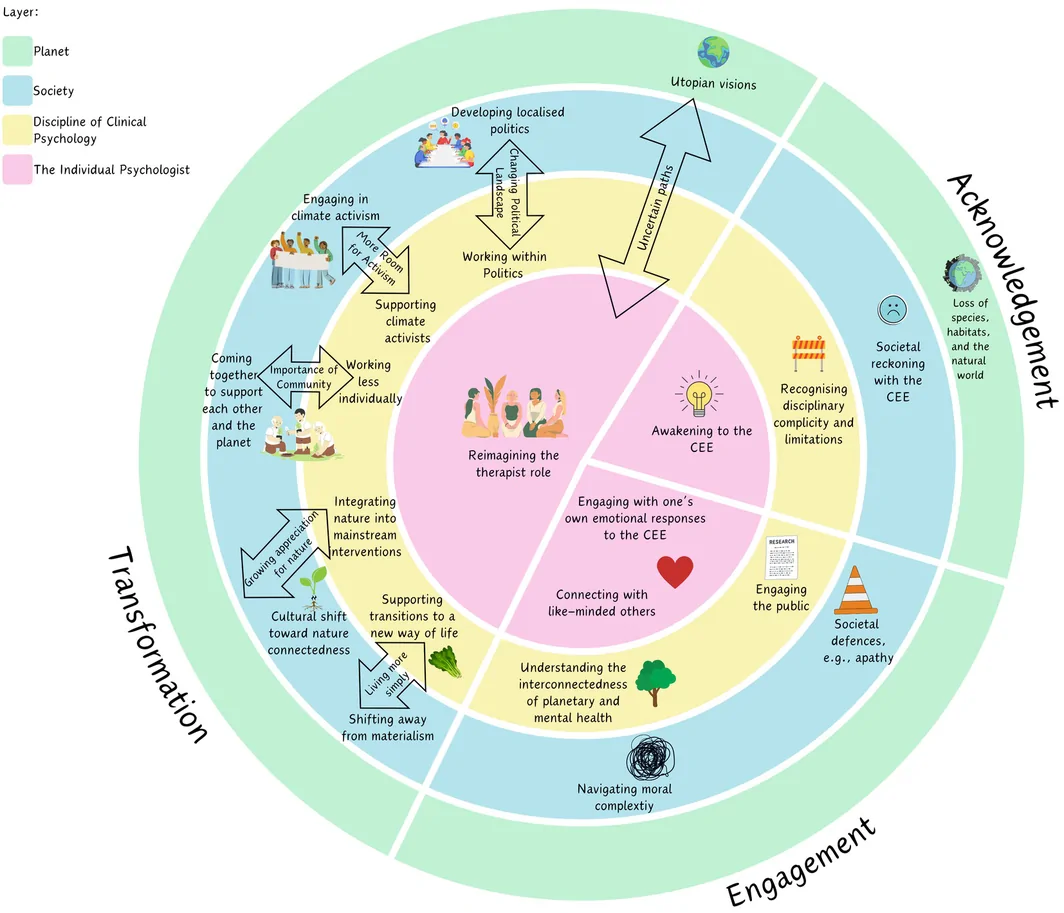
Thematic map across the four ecological layers.

### Engagement

Most stories could not contemplate engaging in change until the negative aspects of the CEE and present‐day society and clinical psychology had been acknowledged, as outlined in the previous theme. However, following the acknowledgement, Ashley and their wider community moved towards engagement with the CEE, which this theme captures. This development echoes the Stages of Change Model, where acknowledgement aligns with ‘Contemplation’ and engagement with ‘Preparation’ (Prochaska et al., 2008). Engagement was represented across all layers of the thematic map. Participants described new ways of understanding mental health, shaped by the CEE and nature connection, across the psychologist, clinical psychology and societal layers.

Within the stories, clinical psychology as a discipline, and Ashley as an individual psychologist, began to recognise planetary health as integral to understanding mental health. PI described Ashley's engagement with the concept of planetary health: ‘She knew at its basic level each human is made from the same compounds as the earth herself, and…realised societies sickness (egoism, disconnect, lack of consciousness) is related to the planets destruction’. This reflects the idea that planetary ill‐health contributes to societal mental ill‐health, aligning with Zelenski and colleagues' findings that nature connection can support pro‐environmental action (Zelenski et al., 2023).

Meeting on an emotional level was deemed important for this engagement, as explained by AW: ‘By talking about her feelings with other like‐minded individuals, Ashley started to find some support. She was able to see a way forward, and what part she could play, she was less pessimistic about the earth's future. She felt hope for the first time in a long while’. Such connection with like‐minded individuals was noted across stories as supporting psychologists' active engagement with the CEE. There was recognition that these emotional impacts affected clients as well as clinicians. EA stated: ‘Almost every client she saw shared the same fears as her, she felt inadequate and lost. How could she help them when she couldn't help herself?’ This highlights the relational dimensions of climate distress, resonating with research on relational resilience, which supports the idea that transformation emerges through connection (Adams et al., 2021).

Engagement also extended outward, as participants described Ashley engaging society in the CEE. FV described Ashley as being ‘a voice for people spreading awareness for a better planet’. This external engagement took various forms: starting charities, using social media and promoting research. These align with the claim that psychologists are well placed to support public engagement with environmental issues (Thompson et al., 2023).

Despite stories describing factors that supported engagement in the CEE, others described barriers. Some stories highlighted societal resistance, SC described apathy as a defence mechanism: ‘By '23, societies around the world were beginning to realise that our endless cycle of consumption was bearing fewer and fewer fruits, but the weight of apathy still crushed many an attempt to affect real change’, whilst SN highlighted moral disconnects: ‘Strangely enough lots of people in distress that Ashley worked with got this quicker than the people who still thought they were ok…who thought it was ok to travel by plane, or take a cruise, or drive everywhere, because they worked hard and they deserved it’.

Other stories highlighted moral dilemmas that disrupted engagement. PL described Ashley trying to persuade her family to ‘shell out for an electric car, which they said was too expensive, and relied on batteries using lithium from countries in the global south where local communities had been devastated by lithium mining’. This focus on barriers reflects the notion of a ‘Perfect Moral Storm,’ where complexity and perceived futility can lead to inaction (Gardiner, 2011); and a wider literature addressing the individual, interpersonal and social barriers involved in responding to the CEE (e.g. Norgaard, 2011).

Participants stressed that clinical psychology should engage with the full spectrum of emotional responses to the CEE, highlighting how emotional engagement not only supports clients in understanding their psychological responses to the CEE, but can also lead to deeper understanding and collective motivation, again paralleling claims made in academic literature (e.g. Pihkala, 2024b). LV described how clinical psychology ‘saw that our particular contribution could lie in our understanding of how emotions could help to forge…connections; love of the natural world and our fellow humans—accepting that love entails responsibility for the beloved; grief for the irreparable damage that has been done; anger to spur action and provide courage to face a future without many of the comforts and distractions we had taken for granted’.

In sum, this theme captures the multifaceted ways participants imagined engaging with the CEE as a critical part of creating a role for clinical psychology within it. Stories included psychologists engaging personally, forming connections with others, validating distress and supporting wider societal engagement—despite facing resistance and moral complexity.

### Transformation

Having acknowledged and engaged with the CEE, many participants imagined a future where clinical psychology actively contributes to mitigation and adaptation efforts. These visions rarely positioned clinical psychology in isolation; instead, its evolution was seen as entangled with broader societal and planetary shifts. As KD's story attested to, what made a difference was ‘forming good and genuine foundations in trust, truth and togetherness both within ourselves, with each other, and with the natural living earth…’.

This emphasis on togetherness underscores how clinical psychology's imagined role was not as a separate agent of change, but as part of a broader, collective transformation. The superordinate theme Transformation captures this interconnected evolution across individual, professional, societal and planetary layers.

The subthemes that follow reflect key dimensions of this transformation: Living More Simply, Growing Appreciation for Nature, Importance of Community, More Room for Climate Activism and Changing Political Landscape. A final subtheme, Uncertain Paths, captures the tensions, unknowns and tentative hope that remained—reminding us that transformation is not linear and shaped by competing forces within and beyond clinical psychology.

#### Living more simply

This subtheme captures participants' vision of society moving away from materialism and overconsumption towards simpler living. Psychologists were imagined as supporting this transition—both individually and collectively—by helping people adjust to new ways of life and ‘really see’ through critical consciousness raising.

Stories described improved wellbeing from these lifestyle changes. WK described how ‘Sacrifices were made—travel and leisure time are very different now, but wellbeing improvements far outweigh these costs’. AW added, ‘Everyone was happier—once they had adjusted to not having the latest phone, or new car, or clothes they would only wear once’. Despite these improvements to wellbeing, some stories acknowledged the cultural resistance associated with such a transition, where consumerist values and entitlement were presented as barriers to change. PL described how Ashley ‘became vegan, and tried to persuade her parents to, but they said they were too attached to eating red meat…also they refused to stop flying, saying they wanted to travel the world before they were too old to see it’.

Stories imagined psychologists using their therapeutic skills to support people in navigating these changes. For example, LV described how psychologists ‘found that our skills in helping people to make transitions, to think more flexibly, and let go of imperatives that were no longer useful or relevant in a time of great flux were useful’. Whilst WK explained that ‘Psychologists across the world used values‐based approaches to help people connect with things most meaningful to them…this meant that people naturally began to rely less on acquisition of resources, comparison, and ultimately led to the end of capitalist societies’. This aligns with Williams and Samuel's (2024) paper on Acceptance and Commitment Therapy (ACT) as a tool to support climate distress through values‐led action.

Participants also highlighted the importance of raising collective awareness. TE remarked on psychology's role in helping people ‘see’, and PI imagined that ‘If we can increase people's awareness/consciousness, so too will we raise the consciousness of the planet and have a chance to bring civilisation and non human life back from the brink of collapse’. In some stories Ashley was portrayed as having a key role in this shift, as UR described: ‘Ashley felt relieved at having been able to influence this shift in collective consciousness and proud of all the ways they and many others had worked together to overcome harmful systems’.

Across these stories, participants suggested psychologists helped support both the emotional and cognitive work needed to enable simpler, more sustainable lives, with critical consciousness work cited as pivotal to broader social and planetary transformation.

#### Growing appreciation for nature

This subtheme captures how a societal shift towards simpler living fostered a deepened connection with nature, reflected not only in everyday practices but also in how psychologists imagined embedding nature connection into mental health work.

On a societal level, many stories described a cultural change in how humans relate to the natural world. LS wrote ‘We no longer believed that we owned these soils … but they in fact owned us and we were a part of the natural system. We nurtured the land and seas and rivers and skies and found great comfort’. With this change, wellbeing was enhanced through engaging with nature‐based practices, such as gardening, outside yoga and growing vegetables. BA described ‘People who supported community gardens, bought natural products and grew their own vegetables all appeared to have better mental health’. These story excerpts echo research establishing associations between connection to nature and happiness and wellbeing (Capaldi et al., 2014).

As society embraced nature connection in participants' stories, it was commonly deemed even more necessary for psychologists to draw upon it in therapy. Participants imagined psychologists offering things like ‘gardening, growing vegetables, planting trees’ (EP), an ‘outdoor mindfulness and movement group’ (CE) and ‘therapeutic walks’ (DI) as a means to benefit both people and the planet. NL described this as creating an ‘Enormous shift in their wellbeing… and even better, in the markers of planetary health’, reflecting growing evidence that nature connection promotes human wellbeing and environmental stewardship (Barragan‐Jason et al., 2023).

Psychologists were also imagined as spreading these ideas more widely by delivering ‘teaching and training to a variety of groups about the importance of nature connection and mental wellbeing’ (EP) as well as educating ‘children and young people to prevent “eco‐anxiety”’ (KW) through early intervention. Such visions resonate with evidence that nature exposure supports both pro‐environmental behaviours and mental health across age groups (Pensini et al., 2016; Tillmann et al., 2018).

#### Importance of community

This subtheme reflects the central role participants gave to collectivist cultures within the stories—emphasising the value of connection, shared responsibility and relational ways of working, both in society and within clinical psychology.

Many stories described a move away from individualism towards communities bound by mutual support and care. Stories depicted community‐based and co‐operatively owned community infrastructure, where wealth was redistributed through these channels. Group 1 suggested that ‘Food was shared, transport was shared, people opened their gardens, rich people gave’ and LV described that ‘The task of survival was shared by all’. Such visions echo Hofstede's (1994) argument that in collectivist cultures, ‘relationship prevails over task’. WK described a ‘culture of compassion’ and other stories depicted increases in tactile affection: ‘Suddenly people started to hug in the streets’ (Group 1).

This shift towards a relational way of being also appeared within clinical psychology, where compassion was central to the profession. VN described ‘We found our common humanity in loving the natural world and brought it into our workplaces’. Furthermore, the profession was reimagined as being deeply embedded within communities, where ‘Neighbourhood psychologists become regular and normal parts of communities’, (Group 6). This was taken further with a shift towards clinical psychology engaging with community psychology approaches. JI described Ashley ‘meeting attenders at local community voluntary sector organisations… hearing the stories of people there’, whilst UC reflected that such approaches enabled Ashley ‘to empower, rather than pathologise, these communities, to co‐produce solutions to the emergency they had faced’. These visions reflect core principles of community psychology—working alongside others, empowering communities and addressing systemic barriers to action (Thompson et al., 2022).

Participants frequently imagined psychologists delivering group‐based interventions, since ‘That fitted better the ethos of the times’ (LV). Group 5 referenced ‘Climate Fresk’; whilst others mentioned ‘climate cafés’ (SI) and the ‘Work That Reconnects’ (UR). Such community‐based approaches were described as protective in the face of climate‐related distress. FN described that this helped ‘us address feelings of guilt and shame… many of [us] came out the other side invigorated and with a deeper understanding of what we had to do in our often desperate struggles’. Such accounts suggest that collective processing of climate distress offers emotional transformation and highlights the power of such solidarity. As Riemer and Harré (2017) suggest, community psychology can support the creation of transformative change through collaboration, empowerment and systems understanding.

#### More room for climate activism

This subtheme captures how participants envisioned both society and clinical psychology embracing activism as a legitimate and necessary response to the CEE.

Activism was widely depicted as a collective force—‘a coming together of communities who stood against the perpetrators, the enablers, and the divisive media narratives’ (NZ)—that included protests, strikes, petitions and public campaigns. It was imagined that a growing collaboration between psychologists and climate movements strengthened climate activism. FN described that ‘Some of the deep capacity and resilience of our movement owed a lot to what we were able to work out with the support of the increasing numbers in psychology professions who were in active solidarity with our movement’. Furthermore, participants described the psychological benefits of activism, for example, ‘Slowly I realised I felt better just standing with everyone. And I learned things’ (IE).

Some stories depicted narratives of psychologists becoming activists themselves. SI described a dramatic shift: ‘The turning point came when first tens, then hundreds, then thousands of risk‐averse and cautious Clinical Psychologists put their liberty on the line in successive, growing mass protests’ and Group 3 took this further: ‘Ashley and some psychologists kind of pioneer a complete like rejection and rebellion against… professional bodies… unless climate is central… [Audience cheers, one member says ‘fantastic’]’.

Participants also described psychologists supporting activists' wellbeing and identity formation. WK imagined therapists helping activists connect to their inner strength: ‘Psychologists… helped them to access their power to harness humans' innate goodness and trust that their actions would be enough’. This aligns with research suggesting that supporting the formation of a group identity may help reduce climate distress and enhance collective efficacy (Williams, 2023).

Others envisioned psychologists fostering regenerative activist cultures, such as psychologists advising ‘the activist groups on how to be caring and supportive of each other’ (YS) and AM added that ‘[Ashley helped] activists experiencing climate grief… she learned about the mental stress that activists experience because they feel responsible for the future of the world’.

This emotional toll of activism is well documented (e.g. activism burnout, eco‐grief; Holthaus, 2023), and participants foresaw clinical psychologists supporting both the psychological and strategic sustainability of activist movements.

#### Changing political landscape

Across some stories, participants imagined a future in which both society and clinical psychology took on more political roles—either by directly influencing policy or through more participatory, devolved leadership structures.

Many stories envisioned psychologists working closely with government, with one even describing a ‘government department…for Clinical Psychology and World Confidence’ (WP). Other political roles for psychologists ranged from ‘a clinical psychologist advisor in government’ (BM) to leading ‘reflective practice for the UK government’ (Group 6) or running ‘a support group for MPs’ (IE). Participants saw psychologists as uniquely equipped to support political change, helping leaders confront distress and act more empathetically. EP imagined psychologists using ‘their positions of power, empathy and communication…to help them [MPs] turn towards their own distress… and to fight for change’. These accounts echo wider debates about psychology's neutrality. Rahim and Cooke (2019) argue that neutrality may amount to complicity with harmful policy, and that psychologists must speak out when mental health is at stake.

Whilst clinical psychology was imagined becoming more politically engaged, society was seen to move towards devolved power—e.g., through citizens' assemblies or grassroots decision‐making. WK described that ‘Community leaders came together to decide national and international agendas… funds and resources were evenly distributed’ (WK). This revealed a tension: clinical psychology's imagined next step was to become more political within existing structures, whilst society's imagined next step was to dismantle them.

Community psychology was repeatedly presented as a potential bridge—offering a way for psychologists to engage politically whilst supporting power‐sharing and co‐production. KD imagined clinical psychology co‐creating ‘a purer and more participatory paradigm of practice…which… became stronger and ever more sustaining’. This vision aligns with Riemer and Harré's (2017) call for psychologists to ‘enter the deeply democratic process of working with others to discuss how we can live together well on this planet’ (p. 449).

#### Uncertain paths

Many participants expressed hope for a better future, but struggled to describe the precise systemic shifts needed to achieve these prospects. Vague language was common, such as ‘Humanity was mobilised on a personal, local, national and global basis’ (AW). Furthermore, there was often a sense of waiting for systemic change to happen before fully acting; for example, KF described that ‘All Clinical Psychology as a profession could do was to be ready to help build the alternative future when national/international policy eventually responded to the climate emergency’. This reflects a kind of passive readiness rather than detailed transformation planning. Whilst these stories showed desire and emotional resonance, some lacked clarity on the pathway to change.

Despite this uncertainty, many narratives ended with uplifting utopian visions marked by emotional relief and improved mental health. GW's vivid portrayal encapsulated this collective hope:

‘Nature roared as it made its recovery… The beauty of nature was instilled within humans… and that beauty transformed mental health. With people breathing cleaner air, they exhaled clearer thoughts… There wasn't traffic tormenting the roads of people's brains… An inner peace was there and it healed the scars of the world and the very beings that inhabited the planet’.

Such imagery suggests that for many, imagining healing planetary systems and improved mental health were inseparable. Though sometimes lacking a roadmap, the stories offered an emotional anchor: peace, clarity and reconnection—with each other and the Earth.

## DISCUSSION

This study used story completion to explore how clinical psychology might mitigate and adapt to the CEE. Three overarching themes emerged: Acknowledgement, Engagement and Transformation. These themes describe a psychological process echoing the value‐belief‐norm model (Stern et al., 1999), where individuals become aware that valued entities are under threat, feel a sense of moral obligation to address this, and ultimately take action. Participants' stories also introduced an important emotional element—grief—which catalysed movement from awareness to engagement. This aligns with the Active Hope model, in which grief must be honoured before hopeful action can occur (Macy & Johnstone, 2022), and meaning reconstruction models of grief, where individuals integrate loss into their evolving worldview (Neimeyer et al., 2014).

Across all three themes, participants imagined multiple roles for clinical psychology. In the Acknowledgement theme, psychologists were seen as supporting recognition of both the roots of the CEE and the emotional responses it evokes. Current approaches such as Climate Cafés (Calabria & Marks, 2024) and The Work That Reconnects (Macy & Johnstone, 2022) provide spaces for this, and psychologists are well placed to facilitate individual and collective grief work (Cunsolo & Ellis, 2018).

From this process of acknowledgement, participants imagined psychologists engaging in alternative conceptualisations of mental health, creating emotionally safe spaces and using their understanding of defences and denial to support climate engagement. These responses suggest a move towards a systems‐based approach to distress, consistent with ecological models of health (Bronfenbrenner, 1979), which view mental health as arising from nested environmental, community and interpersonal factors.

In the final theme, Transformation, the roles of psychologists were envisioned as changing alongside changes in the discipline of clinical psychology and broader societal transformations towards living more simply and a growing appreciation for the natural world and community. These imagined futures resonate with emerging evidence that demonstrates how nature‐based interventions can effectively improve mental health and wellbeing (Coventry et al., 2021; Menhas et al., 2024). Delivering such work would align with psychologists' public health duties (HCPC, 2023) and with the growing recognition that mental health and planetary health are interdependent. However, it is important to note that further research is needed into the effectiveness of nature‐based interventions in comparison to more traditional talking therapies.

Participants also imagined psychologists becoming more politically engaged, shaping policy, supporting activist wellbeing and challenging systemic causes of distress. These roles align with community psychology principles and mirror contemporary calls for training that includes political literacy and socio‐ecological advocacy (Turner, 2023). This echoes findings from Williams et al. (2024), who highlighted that mental health professionals working with young people can feel unprepared and unsupported in responding to climate distress, due in part to a lack of service‐level frameworks or professional guidance. In the UK, the new accreditation standards for doctoral clinical psychology training call on programmes to address sustainability and planetary health, public and preventative health, and systemic influences on wellbeing (British Psychological Society, 2025), reinforcing the professional obligation to respond to the CEE. Such professional shifts reflect broader identity transitions, where psychologists adapt to shifting cultural and environmental demands. This supports theories of professional identity development as dynamic and values‐driven (Trede et al., 2012).

Methodologically, this study demonstrates the potential for story completion to access unspoken assumptions, emotional undercurrents and role tensions that shape practice. Unlike traditional interviews, story completion taps into imaginative and sometimes conflicted visions of clinical identity. This may offer a useful complement to reflective practice and supervision when exploring emerging roles and ethical frontiers in the profession.

Finally, it is noteworthy that some participants struggled to imagine utopian futures. In contexts such as the CEE, imagination is often normatively constrained—individuals are encouraged to focus on manageable actions, adaptation, or personal responsibility, rather than large‐scale, transformative change (Randall, 2009). This can lead to a kind of narrative foreclosure, where possibilities for radical or utopian futures are implicitly closed off. Supporting imagination—and resisting its erosion—may therefore become a necessary therapeutic goal in itself.

Whilst these imagined roles challenge the status quo of clinical psychology, it is perhaps unethical to help individuals adapt to a world order that perpetuates psychological harm (Watkins & Shulman, 2008). In line with this, our paper proposes that clinical psychology must evolve towards a model of ecological reflexivity, where therapeutic work is conducted not just in the service of individual wellbeing, but in solidarity with communities and ecosystems.

## CONCLUSION

This study presents a vision of how clinical psychology might contribute to mitigating and adapting to the CEE, grounded in collective, nature‐based and socially engaged practices. Through imagining future roles, participants articulated how clinical psychology could evolve at the level of both the individual psychologist and the discipline as a whole. Their imagined futures often resonated with existing literature, reinforcing the relevance—and feasibility—of many of the proposed directions.

Informed by participants' accounts and aligned with the three overarching processes of Acknowledgement, Engagement and Transformation, the following are offered as key areas for clinical psychology to consider:

- Supporting planetary health and applying community psychology principles to uphold the profession's preventative and systemic responsibilities.
- Facilitating climate‐related grief work, including models like *Climate Cafes* and *The Work That Reconnects*.
- Delivering nature‐based therapies, training and workshops, and embedding these within services.
- Conducting critical consciousness‐raising work to help individuals and groups understand their roles in the CEE.
- Embracing explicit political engagement, including influencing policy and supporting systems change.

Future research should examine these domains more closely, assessing their acceptability, efficacy and sustainability in clinical contexts. Research practices themselves must also model sustainability, reflecting psychology's role in climate adaptation and mitigation.

Finally, this study demonstrates the potential of story completion methods in co‐creating imagined futures. Participants showed that meaningful action often followed a process of acknowledging reality, engaging emotionally and cognitively and envisioning transformative change. The imagined futures presented here are not naïve utopias; they are grounded in practice, experience and existing psychological knowledge. With commitment and courage, clinical psychology can be part of reshaping the present—and reimagining the future.

## AUTHOR CONTRIBUTIONS

**Georgia King:** Conceptualization; investigation; formal analysis; visualization; project administration; writing – original draft; writing – review and editing. **Matthew Adams:** Conceptualization; methodology; supervision; writing – review and editing. **Nick Maguire:** Conceptualization; supervision; writing – review and editing. **Marc Williams:** Conceptualization; methodology; supervision; writing – review and editing.

## CONFLICT OF INTEREST STATEMENT

Marc Williams was one of the Editors‐in‐Chief of Psychology and Psychotherapy: Theory, Research and Practice at the time of submission. To avoid any potential conflict of interest, he had no involvement in the peer‐review process or editorial decision‐making for this manuscript.

## Supporting information


Table S1.

## Data Availability

Research data are provided as excerpts in the manuscript. Transcripts from the participant interviews and focus groups are not shared.
