# Supplementary material for: Reimagining clinical psychology for a changing planet
Source: Psychol Psychother. 2026 May 14;99(4):1309–28. doi: 10.1111/papt.70050 (PMC13749241; doi:10.1111/papt.70050)
Supplement: Supplementary file 1 [file PAPT-99-1309-s001.docx]

**Table S1. Participant Demographics.**

| Participant pseudonym initials | Practitioner Psychologist/  Climate Activist | Gender | Age | Country of residence | Ethnicity |
| --- | --- | --- | --- | --- | --- |
| LV | Clinical Psychologist | Female | 77 | UK | White - British |
| JI | Clinical Psychologist | Female | 69 | UK | White - British |
| KB | Climate Activist | Female | 23 | Indonesia | Javanese |
| UC | Trainee Clinical Psychologist | Female | 31 | UK | White - British |
| NZ | Trainee Clinical Psychologist | Male | 31 | UK | White - British |
| GT | Clinical Psychologist | Male | 43 | UK | White - British |
| SI | Clinical Psychologist | Female | 37 | UK | White - British |
| VN | Clinical Psychologist | Female | 56 | UK | White - British |
| KW | Trainee Clinical Psychologist | Female | 26 | UK | White - British |
| BM | Trainee Clinical Psychologist | Female | 35 | UK | White - British |
| CE | Trainee Clinical Psychologist | Female | 29 | UK | White - British |
| YH | Climate Activist | Female | 70 | UK | White - British |
| DA | Forensic Psychologist | Female | 34 | UK | Greek |
| NL | Trainee Educational Psychologist | Female | 51 | UK | White - other |
| HS | Trainee Clinical Psychologist | Female | 27 | UK | White - British |
| PI | Trainee Clinical Psychologist | Female | 32 | UK | White - British |
| FN | Climate Activist | Male | 70 | UK | White - British |
| LK | Trainee Counselling Psychologist | Female | 27 | UK | Mixed - White Asian |
| SC | Trainee Educational Psychologist | Other | 32 | UK | White and Southeast Asian |
| IE | Climate Activist | Female | 66 | UK | White - British |
| WP | Climate Activist | Female | 62 | UK | White - British |
| SN | Climate Activist | Female | 62 | UK | White - British |
| TE | Climate Activist | Male | 67 | UK | White - British |
| GV | Climate Activist | Male | 61 | UK | White - British |
| JA | Climate Activist | Female | 18 | UK | White - British |
| YS | Climate Activist | Female | 75 | UK | White - British |
| AW | Climate Activist | Male | 64 | UK | White - British |
| LS | Climate Activist | Female | 59 | UK | White - British |
| KF | Clinical Psychologist | Female | 66 | UK | White - British |
| BA | Trainee Educational Psychologist | Female | 30 | UK | White - British |
| WK | Clinical Psychologist | Female | 36 | UK | White - British |
| FB | Climate Activist | Male | 71 | UK | White - British |
| PL | Climate Activist | Female | 44 | UK | White - British |
| EP | Trainee Clinical Psychologist | Female | 32 | UK | White - British |
| DI | Climate Activist | Female | 26 | UK | White - British |
| SR | Educational Psychologist | Female | 62 | UK | White - British |
| AG | Climate Activist | Male | 24 | UK | White - British |
| UR | Climate Activist | Female | 38 | UK | White - British |
| RO | Climate Activist | Male | 73 | UK | White - British |
| KD | Educational Psychologist | Other | 50 | UK | Prefer not to say |
| NC | Climate Activist | Male | 19 | UK | Polish |
| GW | Climate Activist | Male | 19 | UK | White - British |
| FV | Climate Activist | Female | 18 | UK | White - British |
| AM | Climate Activist | Female | 18 | UK | Black - African Caribbean and White- British |
| OV | Climate Activist | Female | 18 | UK | Asian - Chinese |
| EA | Climate Activist | Female | 19 | UK | White - British |
| PA | Climate Activist | Female | 18 | UK | White - British |
| BL | Climate Activist | Female | 21 | UK | White - British |
| TW | Climate Activist | Female | 19 | UK | White - British |
